# D-Tagatose: A Rare Sugar with Functional Properties and Antimicrobial Potential against Oral Species

**DOI:** 10.3390/nu16121943

**Published:** 2024-06-19

**Authors:** Adriana de Cássia Ortiz, Simone Ortiz Moura Fideles, Carlos Henrique Bertoni Reis, Bruna Trazzi Pagani, Lívia Maluf Menegazzo Bueno, Matheus Bento Medeiros Moscatel, Rogerio Leone Buchaim, Daniela Vieira Buchaim

**Affiliations:** 1 Department of Biological Sciences, Bauru School of Dentistry (FOB/USP), University of Sao Paulo, Bauru 17012-901, Brazil; adrianacsortiz@gmail.com (A.d.C.O.); simoneortizf@gmail.com (S.O.M.F.); dr.carloshenriquereis@usp.br (C.H.B.R.); brunatrazzi@usp.br (B.T.P.); matheusbmm.96@usp.br (M.B.M.M.); 2 Postgraduate Program in Structural and Functional Interactions in Rehabilitation, University of Marilia (UNIMAR), Marília 17525-902, Brazil; danibuchaim@alumni.usp.br; 3 Dentistry School, University of Marilia (UNIMAR), Marília 17525-902, Brazil; 4 Dentistry School, University Center of Adamantina (UNIFAI), Adamantina 17800-000, Brazil; likamaluf@usp.br; 5 Graduate Program in Anatomy of Domestic and Wild Animals, Faculty of Veterinary Medicine and Animal Science (FMVZ), University of Sao Paulo (USP), Sao Paulo 05508-270, Brazil; 6 Medical School, University Center of Adamantina (UNIFAI), Adamantina 17800-000, Brazil

**Keywords:** tagatose, sweetening agents, dietary carbohydrates, metabolic diseases, diabetes mellitus, biofilms, dental caries, oral health

## Abstract

Carbohydrates have a dietary role, but excessive consumption of high-calorie sugars can contribute to an increased incidence of metabolic diseases and dental caries. Recently, carbohydrates with sweetening properties and low caloric value, such as D-tagatose, have been investigated as alternative sugars. D-tagatose is a rare sugar that has nutritional and functional properties of great interest for health. This literature review presents an approach to the biological effects of D-tagatose, emphasizing its benefits for oral health. Studies report that D-tagatose has antioxidant and prebiotic effects, low digestibility, reduced glycemic and insulinemic responses, and the potential to improve the lipid profile, constituting an alternative for diabetes mellitus and obesity. It can also be observed that D-tagatose has an antioxidant action, favoring the elimination of free radicals and, consequently, causing a reduction in cellular oxidative stress. Furthermore, it also has antibacterial potential against oral species. Regarding oral health, studies have shown that D-tagatose efficiently reversed bacterial coaggregations, including periodontopathogenic species, and impaired the activity and growth of cariogenic bacteria, such as *S. mutans*. D-tagatose significantly inhibited biofilm formation, pH decrease and insoluble glucan synthesis in *S. mutans* cultures. Salivary *S. mutans* counts were also significantly reduced by the consumption of chewing gum containing D-tagatose and xylitol. In addition, there is evidence that tagatose is effective as an air-polishing powder for biofilm decontamination. The literature indicates that D-tagatose can contribute to the prevention of systemic diseases, also constituting a promising agent to improve oral health.

## 1. Introduction

Carbohydrates have an important role in the diet; however, excessive consumption of high-calorie sugars, such as sucrose, can contribute to an increased incidence of dental caries and metabolic diseases, such as diabetes mellitus. Currently, carbohydrates with sweetening properties and low caloric value have been investigated as substitutes for sucrose, including D-tagatose [1]. D-tagatose is considered a rare sugar because it is found in small quantities in nature, as well as D-allulose or D-psicose, D-allose and several other sugars [1,2]. Interestingly, D-tagatose can be isolated from the gum of *Sterculia setigera*, a species of tree with medicinal properties, and from the lichen species *Roccella* [3,4,5]. Among dietary components, D-tagatose is mainly present in dairy foods and some fruits, such as apples and oranges [3,6,7,8]. In addition to the diet, D-tagatose can be obtained industrially from lactose, through an enzymatic hydrolysis process [6,9]. D-tagatose (C_6_H_12_O_6_) is a ketohexose structurally similar to D-fructose [1], with a crystalline, white and inodorous solid appearance [3]. The chemical structures between these sugars are distinguished by the spatial configuration of the hydroxyl on the fourth carbon, so that D-tagatose constitutes a stereoisomer (epimer) of D-fructose [3,6,10]. From a nutritional perspective, D-tagatose has several advantages over caloric sugars. 

D-tagatose is a sweet-tasting monosaccharide that has important nutritional properties. In terms of taste, D-tagatose has a relative sweetness of about 92% compared to sucrose [3,6,10,11,12,13]. However, unlike sucrose (4 kcal/g), D-tagatose is a low-calorie sugar, and its energy value is estimated at approximately 1.5 kcal/g (38%) [7,11,12]. In addition to its low caloric contribution, D-tagatose has good palatability, high solubility and adequate bulking properties, presenting applicability as a flavor enhancer in beverages and dairy products, such as soft drinks and yogurts [1,12]. Like some sugars, D-tagatose participates in the Maillard reaction, promoting the browning of foods [3]. Currently, D-tagatose has been used as a sweetener, humectant, texturizer or stabilizer in a variety of low-calorie foods. Its applications in the food industry include soft drinks, cereals, chewing gum, chocolate, sweets, caramels, yogurts, ice creams, nutritional supplements and dairy products, among others [3,6,14].

The nutritional value of D-tagatose is related to the biological properties of this sugar. D-tagatose is a sugar that is poorly absorbed by the organism, which contributes to its low energy content [1]. About 20% of consumed tagatose is absorbed by the small intestine, with most of it fermented by colonic bacteria into short-chain fatty acids, which are almost completely absorbed, and into gaseous products [1,3,5,6,7,12]. In general, D-tagatose is well tolerated, with no significant adverse effects associated with dietary use. However, some mild or moderate gastrointestinal events, such as nausea, diarrhea, flatulence and intestinal distension, have already been reported due to the consumption of this sugar, which has been associated with the fermentation of the unabsorbed fraction [15,16,17]. Furthermore, according to the literature, exposure to D-tagatose did not cause genotoxic or teratogenic changes, or clinically relevant alterations [18,19,20,21]. The safety of the dietary use of D-tagatose has been evaluated in studies with animal models and in clinical trials and, due to the negative results of genotoxicity and toxicity tests, in 2001, D-tagatose was recognized by the Food and Drug Administration (FDA) as GRAS (Generally Recognized As Safe), being widely used as a low-calorie nutritive sweetener (GRAS–GRN 000078) [3,6,11,14]. In addition, D-tagatose has been approved as a “new food ingredient” by the European Union, without any restrictions on its use [6,14,22]. The FAO/WHO Joint Expert Committee on Food Additives (JECFA) also recognized that D-tagatose was not genotoxic, embryotoxic or teratogenic. Thus, D-tagatose has been considered safe for use in foods, pharmaceuticals and cosmetics. This literature review presents an overview of the functional and antimicrobial properties of D-tagatose and its impacts on systemic and oral health.

## 2. Biological Effects of D-Tagatose

### 2.1. Functional Properties of D-Tagatose and Its Role in Systemic Health

In addition to its considerable nutritional value, D-tagatose has functional properties that can benefit health [14]. According to the literature, D-tagatose has an antioxidant effect, prebiotic potential, low digestibility and reduced glycemic and insulinemic responses. There are reports that D-tagatose can exert antioxidant action through the elimination of free radicals, thus reducing cellular oxidative stress [9,23,24,25,26]. The prebiotic properties of D-tagatose favor the growth of intestinal microflora, considering that the majority of ingested D-tagatose is fermented in the colon by resident bacteria [9,23,24,25,27]. Another important characteristic of D-tagatose is related to the reduced glycemic and insulinemic responses associated with the consumption of this sugar. Unlike sucrose, the metabolization of D-tagatose does not significantly interfere with glucose levels, being considered an interesting alternative for use in controlling diabetes mellitus and obesity [6].

Preclinical and clinical studies that investigated the effect of the oral administration of D-tagatose on glucose and insulin levels showed promising results. A clinical trial that investigated the effect of oral administration of D-tagatose (75 g) showed that this sugar, alone, did not alter glucose and insulin levels in healthy or diabetic individuals. In addition, this study reports that the administration of D-tagatose, associated with previous glucose intake, did not significantly alter insulin levels, and also significantly minimized the increase in glucose levels in diabetic individuals, in a dose-dependent manner. This study highlights that D-tagatose can act by reducing glucose absorption, thus constituting a promising dietary agent to help control diabetes mellitus [15]. A subsequent study performed to analyze the tolerance and metabolic effects of D-tagatose (15 g/3 times a day) in subjects with type 2 diabetes showed that oral administration of this sugar led to significant improvements in body weight and high-density lipoprotein levels, in addition to a non-significant reduction in glycated hemoglobin. Furthermore, D-tagatose was well tolerated, with few reports of mild and transient gastrointestinal events [28]. Ensor and colleagues (2014) also reported that D-tagatose administered orally in different dosages (2.5, 5 and 7.5 g) was well tolerated among individuals with type 2 diabetes who consumed sugar daily (3 times a day) for 6 months. In this study, the consumption of D-tagatose reduced the blood levels of glycated hemoglobin (HbA1c) and fasting blood glucose, in addition to causing a reduction in average body weight in a dose-dependent manner. The highest dosage tested in the study presented the best results in most of the parameters evaluated [29]. In another clinical trial conducted with diabetic individuals (type 2), the administration of D-tagatose (15 g/3 times a day) resulted in a significant reduction in HbA1c in relation to placebo, but no changes were found in triglyceride and high-density lipoprotein levels (HDL) between groups [30]. A systematic review of the literature conducted by Noronha et al. (2018) found moderate evidence between the effects of administering small doses of fructose and tagatose (10 g) and the reduction in HbA1c and fasting glycemia, emphasizing that future long-term clinical trials will be essential to better support these estimates [31]. 

In general, studies in the literature indicate that D-tagatose may have the potential to improve the lipid profile and glycemic levels, including postprandial glycemia, as well as to benefit the intestinal flora and reduce the expression of pro-inflammatory cytokines [2,32]. However, research must advance to clarify the mechanisms by which D-tagatose interferes with these metabolic processes [2]. One of the likely mechanisms of action of D-tagatose may be related to interference with the absorption of carbohydrates by the gastrointestinal tract and the transport of glucose [14,33]. Reducing carbohydrate absorption can increase satiety, contributing to weight loss [10]. Studies also report that D-tagatose promotes an increase in glycogen synthesis, which may contribute to reducing plasma glucose levels [10]. Thus, rare sugars, such as D-tagatose, have proven to be an interesting alternative to replace caloric sugars [34,35]. Studies have invested in the search for new sweeteners, considering that the consumption of a high-calorie diet can contribute to obesity, cardiovascular disorders and other chronic diseases, such as diabetes mellitus, which constitute serious public health problems that affect the world population [2,34]. 

**Figure 1:**
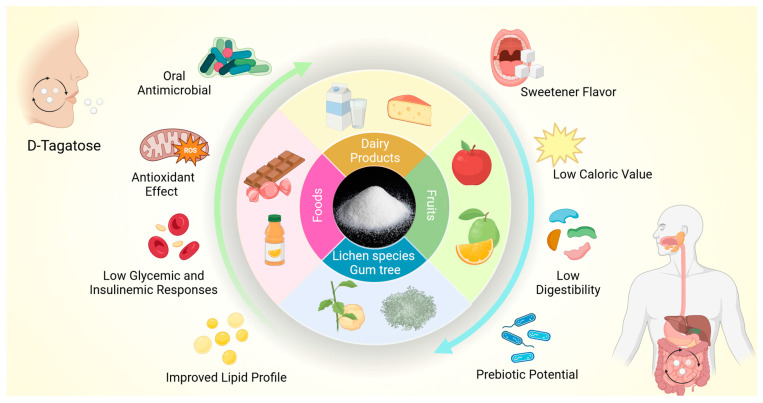
The illustration outlines the main sources of D-tagatose and its nutritional and biological properties. D-tagatose is a rare sugar with a sweet taste and low caloric value. D-tagatose has antioxidant and prebiotic properties, reduced glycemic and insulinemic responses, and the potential to improve the lipid profile, in addition to exerting an antimicrobial effect against oral species.

In this way, the biological properties of D-tagatose can favor systemic health, contributing to the prevention and management of chronic diseases. In addition, D-tagatose can also beneficially impact oral health due to its antibacterial potential [36,37,38,39,40,41]. Figure 1 illustrates the main sources of D-tagatose and its biological properties.

### 2.2. Benefits of D-Tagatose for Oral Health

In addition to its nutritional and functional properties that may contribute to the prevention of systemic diseases, D-tagatose may exert a beneficial effect on oral health. In addition to being considered a non-cariogenic sugar, there is evidence in the literature that D-tagatose may have an antibacterial effect against oral species, including periodontopathogenic and cariogenic strains. This consideration is important because systemic pathologies, such as asthma or allergic rhinitis, can be considered impacting factors for oral health in different age groups [42]. Scribante et al. (2024), through a 6-month randomized clinical study, compared the effectiveness of two remineralizing toothpastes in children suffering from asthma and allergic rhinitis. Forty children aged between 6 and 14 years old with enamel demineralization were evaluated. Using one toothpaste made from zinc hydroxyapatite and the other made from calcium sodium phosphosilicate, they concluded that the tested toothpastes can be proposed for home use in study participants, as they significantly reduced tooth sensitivity and periodontal indices [43]. Therefore, several strategies can improve oral parameters and D-tagatose may represent a promising candidate due to its antimicrobial potential.

According to studies in the literature, D-tagatose can impair bacterial metabolism and the formation of oral biofilms, contributing to the prevention of periodontal disease and dental caries. Thus, considering the role of dietary carbohydrates on the bacterial activity of oral biofilms, D-tagatose may have several advantages over cariogenic carbohydrates, such as sucrose [34,36,37,38,39,40,41]. 

#### Antimicrobial Potential of D-Tagatose against Oral Bacteria

The antimicrobial properties of D-tagatose against oral species have been reported by several studies [36,37,38,39,40,41]. Lu and Levin (2002) investigated the antibacterial potential of different sugars (D-galactose, D-tagatose and D-sorbitol) used to reverse the coaggregation of bacterial species involved in the formation of oral biofilms. Solutions containing D-galactose, D-tagatose or D-sorbitol, at concentrations of 143 to 750 mM, were analyzed on the coaggregation of early and late colonizers of the oral cavity, such as *Streptococcus oralis* (SO34 and C104), *Streptococcus mitis* (J22), *Streptococcus morbillorum* (PK509), *Actinomyces naeslundii* (T14V, PK29 and PK947), *Fusobacterium nucleatum* (PK1594), *Porphyromonas gingivalis* (PK1924), *Prevotella loescheii* (PK1295), *Veillonella atypica* (PK1910), *Capnocytophaga sputigena* (4), *Capnocytophaga ochracea* (25), *Capnocytophaga gingivalis* (27) and *Actinobacillus actinomycetemcomitans* (JP2). Bacterial coaggregation was analyzed visually and measured using a scale from 0 (no coaggregation) to 4 (maximum coaggregation). The study reports that the coaggregations reversed by D-galactose were also sensitive to D-tagatose. D-galactose, at 143 mM, completely or almost completely reversed the bacterial coaggregations; however, higher concentrations of D-tagatose were necessary to reverse these coaggregations. Among the 28 coaggregation pairs established between bacterial species, 60% (17 pairs) were totally or almost completely reversed by D-tagatose. Many species were sensitive to D-tagatose, including streptococci, actinomyces, fusobacteria, porphyromonads and actinobacilli. Thus, treatment with D-tagatose was efficient in reversing coaggregations between early and late colonizers, including Gram-negative species involved in periodontal disease. In contrast, D-sorbitol generally had little effect on reversing bacterial coaggregation. The study highlights the biological potential of D-tagatose to disrupt coaggregations between oral bacteria, emphasizing its importance in controlling periodontal disease. According to the study, D-tagatose may constitute a promising agent for improving oral health [36].

Other studies have investigated the effects of D-tagatose on cariogenic bacteria, such as *Streptococcus mutans*. Sawada et al. (2015) investigated the effects of several sugars (xylitol, D-psicose, L-psicose, D-tagatose and L-tagatose), used at a concentration of 10% (*w*/*v*), on the growth of *S. mutans* (GS5), acid production and insoluble glucan synthesis. This study compared the antimicrobial effects of xylitol, a non-cariogenic sugar, and the ketohexoses tagatose and psicose, which are considered rare sugars due to their low availability in nature. After 12 h of cultivation, all the tested sugars significantly inhibited bacterial growth compared to the control, with D-tagatose showing the best result, statistically differing from xylitol. In the presence of sucrose (1%), all sugars significantly inhibited the decrease in pH compared to the control, with D-tagatose showing the best effect, followed by D-psicose, which differed statistically from xylitol. Regarding the synthesis of insoluble glucan, only xylitol and D-tagatose showed a significant inhibitory effect compared to the control, in the presence of sucrose (1%). Insoluble glucans alter the structure and increase the virulence of biofilms, therefore constituting an important parameter to be analyzed [37].

Hasibul et al. (2018) investigated the effects of D-tagatose, at concentrations of 1 and 4% (*w*/*v*), on the growth of *S. mutans* (GS-5) and on biofilm formation in vitro, as well as on the metabolic activity of this pathogen, assessed through the expression of the enzyme glycosyltransferase B and the synthesis of insoluble glucan. The enzyme glycosyltransferase B participates in the synthesis of insoluble glucans and sucrose is a substrate used by *S. mutans* for the production of these polysaccharides. The study data showed that sucrose (1%), alone, favored the growth of *S. mutans*, promoting a decrease in pH to levels below 5 after 9 h of incubation. In contrast, D-tagatose (1%) delayed the growth of *S. mutans* and the decrease in pH in culture medium supplemented with sucrose (1%). However, no significant differences in bacterial growth were observed between these groups (sucrose and sucrose + D-tagatose) after 24 h of incubation. This study also investigated the effects of different sugars (xylitol, D-glucose, D-tagatose and sucrose) on the formation of *S. mutans* biofilm. *S. mutans* biofilm formation was significantly reduced in medium containing sucrose (1%) supplemented with D-glucose, xylitol, D-tagatose or xylitol + D-tagatose, compared to medium containing sucrose alone (1%). According to the study data, the groups treated with xylitol, D-tagatose or xylitol + D-tagatose showed the lowest biofilm formation, with no significant difference between them. Additionally, the study compared the effect of D-tagatose and xylitol, used in different concentrations (0; 0.5; 1; 2 and 4%), on the formation of *S. mutans* biofilm, in the presence of sucrose (1%). The study reports that D-tagatose showed better results than xylitol, inhibiting biofilm formation in a dose-dependent manner. Scanning electron microscopy analyses also compared the effects of D-tagatose and xylitol, at concentrations of 1 and 4%, on the formation of *S. mutans* biofilm, in the presence of sucrose (1%). After 72 h of incubation, the biofilm quantification analyses showed that D-tagatose significantly reduced biofilm formation, statistically differing from the groups treated with xylitol and the group treated with sucrose alone (1%). Finally, PCR analyses showed that D-tagatose (1%), alone or associated with sucrose (1%), significantly reduced the expression of the glycosyltransferase enzyme, statistically differing from the group treated with sucrose alone (1%). Likewise, D-tagatose (4%) significantly reduced the synthesis of insoluble glucan in the presence of sucrose (1%). Data from this study showed that D-tagatose inhibited bacterial growth and activity, interfering with the use of sucrose by *S. mutans* [38].

The antibacterial potential of D-tagatose against *S. mutans* was also investigated in human saliva samples. Nagamine et al. (2020) counted the total number of bacteria and the number of *S. mutans* colony-forming units (CFU-mL) in samples of stimulated human saliva, collected from 10 adult participants. These samples were cultured in (1) the absence of xylitol or D-tagatose (control), (2) 5% xylitol, (3) 5% D-tagatose and (4) 2.5% xylitol + 2.5% D-tagatose. The results of the in vitro experiments showed that there was a significant reduction in the count of total bacteria and *S. mutans* in the media supplemented with xylitol, D-tagatose and xylitol + D-tagatose, in relation to the control. In both analyses (total bacteria and *S. mutans*), D-tagatose, alone or associated with xylitol, showed a statistically more significant reduction in the count of these bacteria compared to xylitol alone [39]. A randomized clinical trial (double blind) was also conducted to analyze the number of total bacteria, *S. mutans* and lactobacillus (CFU/mL) in the saliva of healthy adult individuals who used chewing gum containing D-tagatose and/or xylitol. The volunteers underwent a preliminary assessment to count the number of *S. mutans* in their saliva. A minimum limit of detection of salivary *S. mutans* (2 × log CFU/mL) was established as an inclusion criterion, so that volunteers who presented counts below this limit were excluded from the study. In addition to the number of salivary *S. mutans*, other inclusion criteria were established, such as absence of diseases, use of antibiotics or immunosuppressants in the last three months, high body temperature, edentulous jaw and use of dentures. Nineteen individuals (21–49 years old) participated in the study, who were divided into three groups (*n* = 6), according to the composition of the chewing gum: (1) 1.5 g D-tagatose, (2) 1.5 g xylitol and (3) 0.75 g D-Tagatose + 0.75 g xylitol (1:1). Participants consumed two tablets of the gum, three times a day (after breakfast, lunch and dinner), for 4 weeks. Stimulated saliva samples were collected at baseline and once a week for a period of 1 month. Variations in salivary flow between individuals were measured. On the same days that saliva was collected, biofilm was collected from the cervical region of the buccal surface of the right lower first molar of each participant. Throughout the experimental period, participants followed their usual oral hygiene routine. Additionally, questionnaires were applied to participants to evaluate various parameters, such as hypersensitivity and digestive problems, among others. Saliva samples were diluted and plated to count the number of total bacteria and *S. mutans*. Regarding the total number of bacteria, the results did not show significant changes over time for all experimental groups, although there was a non-significant trend towards a reduction in bacterial counts, especially for the D-tagatose group. Regarding the *S. mutans* count, only the D-tagatose + xylitol group showed a significant reduction in the number of colonies over time. No significant changes were also reported in the count of salivary lactobacilli for the experimental groups, as well as in the count of *S. mutans* in the biofilm, according to the study data. Furthermore, according to the study, no adverse events were reported due to the consumption of the gums, such as gastrointestinal discomfort, diarrhea, changes in appetite or weight. Unlike in vitro assays, in which D-tagatose alone exerted a significant inhibitory effect on bacterial growth, in the clinical study, an effective reduction in the *S. mutans* population was only obtained by the combination of D-tagatose with xylitol [39].

Analyses of human saliva samples were also carried out by Mayumi et al. (2021). This study performed a metabolomic analysis based on raw data (previous study) obtained by the gas chromatography of unstimulated saliva samples previously collected from 19 individuals, who were also subjected to plaque index analysis. Analysis of the raw data found 114 metabolites, including D-tagatose. The study also reported that there was a significant negative correlation between D-tagatose and the plaque index, suggesting that this sugar may play an inhibitory role on the development of dental biofilm. Additionally, in vitro experiments were performed to investigate the inhibitory potential of D-tagatose on the activity and growth of different strains of streptococci, such as *S. gordonii* (DL1 Challis), *S. mutans* (OMZ175 and UA159) and *S. oralis* (ATCC9811). *S. oralis* is a species commonly found on oral surfaces, while *S. gordonii* is involved in the formation of periodontal biofilm. For cell viability assays, the strains were cultivated in medium containing D-glucose (0.2%), with or without D-tagatose (1 and 5%). The results showed that D-tagatose significantly reduced the viability of *S. gordonii* and *S. mutans*, in a dose-dependent manner. D-tagatose, at a concentration of 1%, promoted an increase in the number of viable cells of *S. oralis*, while it insignificantly reduced the viability of this strain at a concentration of 5%. In experiments conducted to investigate biofilm formation, strains were grown in medium containing sucrose, D-glucose, D-tagatose (0.8%) or no sugar. Analysis using a confocal laser scanning microscope showed that sucrose promoted significant *S. mutans* biofilm formation, while a delicate *S. gordonii* biofilm was observed. In the presence of D-tagatose, there was the formation of insignificant biofilms of *S. gordonii* and *S. mutans*, whereas these strains formed significant biofilms in medium containing D-glucose. Biofilm formation of *S. oralis* was significantly promoted by D-glucose or D-tagatose. To investigate the effect of D-tagatose on the planktonic growth of these bacteria, the strains were cultivated in medium containing D-tagatose or not, at different concentrations (0.1; 0.5; 1; 5 or 10%). At a concentration of 0.5% or higher, D-tagatose delayed the growth of *S. gordonii* and *S. mutans*, while not affecting the growth of *S. oralis* at a concentration of 1% or lower. Higher concentrations of D-tagatose (5 and 10%) inhibited the growth of *S. oralis*, but in smaller proportions compared to other strains (*S. gordonii* and *S. mutans*). Other experiments were also conducted in this study to evaluate the production of extracellular polysaccharide, intracellular metabolites and variations in the gene expression profiles of the bacteria. For this, *S. mutans* (OMZ175) were cultivated in medium containing D-glucose (0.2%), associated or not with D-tagatose (1 and 5%). In the presence of glucose, an increase in extracellular polysaccharide production, in a dose-dependent manner, has been reported. Analysis of the metabolomic profile of the strains revealed that D-tagatose altered the homeostasis of bacterial metabolism. According to the data reported in the study, exposure to D-tagatose resulted in changes in the glycolysis process and alanine metabolism, whose levels were increased in *S. oralis* and significantly decreased in *S. mutans* and *S. gordonii*. In the presence of D-tagatose, the levels of leucine, isoleucine and valine were significantly decreased in cultures of *S. mutans* and *S. gordonii*. On the other hand, D-tagatose promoted a significant increase in lactate in *S. mutans*, without significant changes in *S. gordonii* and, mainly, in *S. oralis*. Gene expression analyses (RNA-Seq Analysis) identified groups of genes differentially expressed in *S. mutans* (116 upregulated and 83 downregulated genes), *S. gordonii* (35 upregulated and 3 downregulated genes) and *S. oralis* (4 upregulated and 8 downregulated genes). According to the analyses performed in the study, the differentially expressed genes were related to several biological processes, such as the phosphoenolpyruvate-dependent sugar phosphotransferase system, lactose metabolism, galactose catabolism, histidine biosynthetic and lactose catabolic processes [40].

The applicability of tagatose as an air-polishing powder for surface decontamination was also investigated. Di Tinco et al. (2021) conducted an in vitro study to evaluate the cleaning capacity and antibacterial potential of glycine and tagatose, used as air-polishing powders on *Pseudomonas aeruginosa* (P1242) biofilm formed on titanium discs. Several powders used in prophylaxis procedures have proven to be efficient as cleaning agents, such as sodium bicarbonate, calcium carbonate and, above all, glycine, considered the gold standard. According to the study, the powder used in prophylactic procedures must have efficient cleaning capacity, which is highly recommended in treatments involving implants, considering that the rough topography of the materials can favor the accumulation of biofilm, compromising the success of the technique. Furthermore, the study highlights that these powders must ideally be biocompatible, considering that the preservation of the vitality and regenerative characteristics of cells and biological tissues are fundamental for an adequate integration process. Thus, to investigate the effects of the tested powders, the *P. aeruginosa* strain was cultivated on titanium discs, in the presence of sucrose (2%). The discs were then subjected to a decontamination process by exposure to glycine or tagatose. The discs in the control group did not receive treatment. The study reports that the *P. aeruginosa* biofilm formed on the titanium discs was significantly reduced after cleaning with air-polishing powders in relation to the control, with a reduction of more than 6 and 4 times after treatment with glycine and tagatose, respectively. The analyses performed to evaluate the persistent biofilm after 30 h of incubation showed that the group treated with glycine showed a 1.7-fold reduction in relation to the control, while the group treated with tagatose did not differ from the control. Analyses that compared the persistent biofilm in relation to the initial biofilm showed that the group treated with glycine showed an 18-fold increase, while the tagatose and control groups showed an increase of approximately 33 times. Despite these differences, according to the study, both glycine and tagatose had efficient cleaning power, although in different magnitudes. In addition to these findings, this study also investigated possible changes in the biological characteristics of stem cells cultured on titanium discs pre-polished with glycine or tagatose. Thus, stem cells derived from the dental pulp of molars of adult individuals were isolated, characterized and cultured for up to 7 days on titanium discs, either unpolished or previously air-polished with glycine or tagatose. Confocal microscopy analyses performed after 7 days of cultivation showed no differences in cell morphology or distribution, and no significant differences were reported with regard to cell adhesion and proliferation. The study also reports that the powders used in the cleaning treatment did not affect the angiogenic potential, measured by the expression of VEGF (vascular endothelial growth factor), as well as the osteogenic potential of the cells, assessed by the expression of the markers RUNX2 (runt-related transcription factor 2) and OCN (osteocalcin). Additionally, scanning electron microscopy analyses conducted to characterize the titanium surface revealed that the powders used in the study did not cause significant changes in the nanotopography of the disc surface. In short, this study concluded that air-polishing treatments with glycine or tagatose presented an effective potential for disc decontamination, without inducing relevant changes in the cellular morphology and nanotopography of the titanium surface [41]. Table 1 summarizes the main outcomes of studies that investigated the biological effects of D-tagatose against oral bacteria.

**Figure 2:**
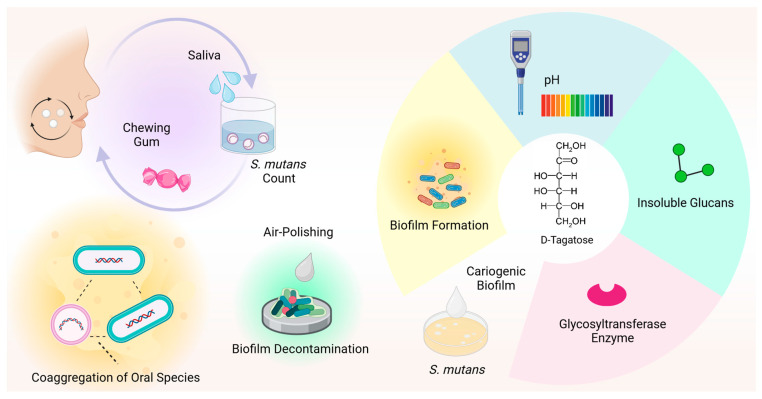
D-tagatose has an antimicrobial effect against oral species. D-tagatose efficiently reversed bacterial coaggregation, including periodontopathogenic species, and significantly inhibited biofilm formation, pH decrease, glycosyltransferase enzyme expression and insoluble glucan synthesis in *S. mutans* cultures. In combination with xylitol, D-tagatose significantly reduced the *S. mutans* count in human saliva. Additionally, tagatose is effective as an air-polishing powder for biofilm decontamination.

Figure 2 provides an overview of the antimicrobial effects of D-tagatose reported in the literature against oral species.

In short, D-tagatose is a rare sugar that combines nutritional and functional properties, presenting dietary applicability as a low-calorie sweetener. D-tagatose also has considerable antibacterial potential against species involved in periodontal disease and dental caries. Thus, the dietary use of D-tagatose can benefit health, considering that excessive consumption of caloric sugars can contribute to the development of chronic diseases. In addition, frequent consumption of cariogenic sugars combined with inadequate oral hygiene constitute factors that increase the risk of developing dental problems, such as caries [44,45,46,47,48]. In this way, the use of oral care formulations containing D-tagatose may constitute a strategy to improve periodontal health and tooth integrity. In terms of oral health, it is important to emphasize that research must advance to expand knowledge about the antimicrobial action of D-tagatose, as well as to investigate the effects of its use in combination with compounds that act on the biofilm or on the tooth structure, such as antiseptics and remineralizing agents, respectively [39,49].

## 3. Conclusions

D-tagatose is a rare sugar with a sweet taste and low caloric value. In addition to the low energy contribution, D-tagatose has other properties of interest for health, such as antioxidant capacity, prebiotic effects, low digestibility, reduced glycemic and insulinemic responses, and potential to improve the lipid profile. According to several studies, the dietary use of D-tagatose is considered safe, constituting an interesting option in conditions of diabetes mellitus and obesity. Thus, D-tagatose has been used in foods, pharmaceuticals and cosmetics. 

In addition to its nutritional and functional properties, D-tagatose is considered a non-cariogenic sugar, which has an antimicrobial effect against oral species. The current literature reports that D-tagatose can disrupt the viability, metabolism and growth of oral bacteria, especially cariogenic strains, such as *S. mutans*. It has been reported that D-tagatose has the ability to interfere with bacterial aggregation, including periodontopathogenic species, and to compromise the formation and virulence of cariogenic biofilms. D-tagatose, alone or in association with other agents, may constitute a promising agent to improve oral health, contributing to the prevention of oral diseases, such as periodontitis and dental caries. However, future research, including preclinical and clinical trials, should be conducted to better elucidate the mechanisms of action of D-tagatose and to expand knowledge about its effects on the metabolism of oral microorganisms, as well as to evaluate the magnitude of its antimicrobial potential in clinical practice.

## Figures and Tables

**Table 1:** Summary of the main outcomes of studies that investigated the biological effects of D-tagatose against oral bacteria.

References	Assay	Intervention	Main Outcomes
Lu and Levin (2002) [36]	In vitro	Solutions containing D-galactose, D-tagatose or D-sorbitol (143 to 750 mM) were tested to reverse the coaggregation of bacterial species related to oral biofilms.	D-galactose and D-tagatose reversed several bacterial coaggregations, while D-sorbitol had little effect. D-tagatose was efficient in completely or almost completely reversing the coaggregation of 17 (60%) of the 28 pairs of coaggregated bacteria, at a concentration of 750 mM or below.
Sawada et al. (2015) [37]	In vitro	The effects of xylitol, D-psicose, L-psicose, D-tagatose or L-tagatose (10%) were evaluated on the growth of *S. mutans* (GS-5), acid production and the synthesis of insoluble glucan.	After 12 h of cultivation, the tested sugars significantly inhibited the growth of *S. mutans*, with D-tagatose showing the best effect, statistically differing from xylitol. The sugars significantly inhibited the decline in pH in the presence of sucrose (1%), with D-tagatose and D-psicose showing the most expressive inhibition, differing statistically from xylitol. Only xylitol and D-tagatose significantly inhibited the synthesis of insoluble glucan, in the presence of sucrose (1%).
Hasibul et al. (2018) [38]	In vitro	The effects of xylitol, D-glucose, D-tagatose and xylitol + D-tagatose on *S. mutans* (GS-5) biofilm formation were compared. The dose–response effect of D-tagatose and xylitol (0; 0.5; 1; 2 and 4%) on biofilm formation was also investigated, as well as the inhibitory potential of D-tagatose on the expression of the enzyme glycosyltransferase B (gtfB) and the synthesis of insoluble glucan.	Xylitol, D-glucose, D-tagatose and xylitol + D-tagatose significantly reduced *S. mutans* biofilm formation in the presence of sucrose (1%). In relation to xylitol, D-tagatose showed better results, inhibiting biofilm formation in a dose-dependent manner. In addition, D-tagatose significantly reduced the expression of the enzyme glycosyltransferase (gtfB) and the synthesis of insoluble glucan in the presence of sucrose (1%).
Nagamine et al. (2020) [39]	In vitro. Randomized clinical trial	In vitro: Oral bacteria from human saliva were cultured in (1) control medium (without xylitol or D-tagatose); (2) 5% D-tagatose; (3) 5% xylitol; (4) 2.5% D-tagatose + 2.5% xylitol. Clinical trial: Experimental groups (*n* = 6): (1) gum containing 1.5 g D-tagatose; (2) gum containing 1.5 g xylitol; (3) gum containing 0.75 g D-tagatose + 0.75 g xylitol. Participants consumed 2 tablets of the gum 3 times/day for 4 weeks. Stimulated saliva and dental biofilm were collected weekly.	In vitro: Xylitol, D-tagatose and xylitol + D-tagatose significantly reduced the total bacterial count and number of colony-forming units of *S. mutans*. D-tagatose, alone or associated with xylitol, showed a more significant reduction in bacterial counts (total bacteria and *S. mutans*), statistically differing from xylitol. Clinical trial: There were no significant changes in the count of total bacteria and salivary lactobacilli, as well as in the count of *S. mutans* in the biofilm. Only the D-tagatose + xylitol group significantly reduced the number of salivary *S. mutans*.
Mayumi et al. (2021) [40]	Salivary metabolome and plaque index analysis. In vitro assays	Analysis of the metabolomic profile of human saliva and plaque index. In vitro assays: Effects of D-tagatose on cell viability (1 and 5%), biofilm formation (0.8%), planktonic growth (0.1; 0.5; 1; 5 and 10%), extracellular polysaccharide production, intracellular metabolites and gene expression (1 and 5%) of *S. mutans*, *S. gordonii* and *S. oralis* strains.	In total, 114 metabolites were identified, including D-tagatose. A significant negative correlation was noted between D-tagatose and the plaque index. In vitro assays: D-tagatose inhibited biofilm formation/planktonic growth and significantly reduced the viability of *S. mutans* and *S. gordonii*. D-tagatose altered the homeostasis of bacterial metabolism and the gene expression profile of the strains. In the presence of glucose, an increase in extracellular polysaccharide production by *S. mutans* has been reported.
Di Tinco et al. (2021) [41]	In vitro	Effects of air-polishing using glycine or tagatose on the formation and persistence of *P. aeruginosa* biofilm on titanium discs. Effects of air-polishing with glycine or tagatose on titanium nanotopography and on the biological properties of human dental pulp stem cells seeded on titanium discs.	Air-polishing with glycine or tagatose significantly reduced the biofilm formed on the titanium discs. Glycine was more efficient than tagatose in reducing persistent biofilm. Air-polishing with glycine or tagatose did not cause relevant changes in the titanium nanotopography, nor did it compromise the biological properties (adhesion, proliferation, morphology, angiogenic and osteogenic potential) of the pulp stem cells.
